# Procalcitonin kinetics after burn injury and burn surgery in septic and non-septic patients – a retrospective observational study

**DOI:** 10.1186/s12871-018-0585-6

**Published:** 2018-09-05

**Authors:** Luís Cabral, Vera Afreixo, Rita Meireles, Miguel Vaz, Margarida Marques, Isabel Tourais, Catarina Chaves, Luís Almeida, José Artur Paiva

**Affiliations:** 1 Department of Plastic Surgery and Burns Unit, Coimbra University Hospital Centre (CHUC), Av. Bissaya Barreto s/n, 3000-075 Coimbra, Portugal; 2 Autonomous Section of Health Sciences (SACS), University of Aveiro, Aveiro, Portugal; 3 CIDMA-Center for Research and Development in Mathematics and Applications; iBiMED-Institute for Biomedicine, University of Aveiro, Aveiro, Portugal; 4 Department of Anesthesiology, Coimbra University Hospital Centre (CHUC), Coimbra, Portugal; 5 Department of Clinical Pathology, Coimbra University Hospital Centre (CHUC), Coimbra, Portugal; 6 MedinUP, Department of Pharmacology and Therapeutics, Faculty of Medicine, University of Porto, Porto, Portugal; 7 Department of Emergency and Intensive Care Medicine, Centro Hospitalar São João, Porto, Portugal; 8 Faculty of Medicine, University of Porto; Grupo de Infecção e Sépsis, Porto, Portugal

**Keywords:** Burns, Sepsis, Procalcitonin, Surgery

## Abstract

**Background:**

Early sepsis diagnosis is crucial for the correct management of burn patients, and it clearly influences outcomes. The systemic inflammatory response triggered by burns mimics sepsis presentation and complicates early sepsis diagnosis. Biomarkers were advocated to aid the diagnosis of early sepsis. Serum procalcitonin (PCT) exhibits fair accuracy and good correlation with sepsis severity, being used in diverse clinical settings. However, few studies have evaluated perioperative changes in PCT levels in burn patients. The present study evaluated PCT kinetics during the first days after burn injury and subsequent surgical interventions to assess PCT utility in distinguishing septic from non-septic inflammatory responses.

**Methods:**

This study was a retrospective observational study of all burn patients admitted to the Coimbra Burns Unit (Portugal) between January 2011 and December 2014 who presented with a total burn surface area ≥ 15% and who underwent subsequent surgery. PCT kinetics were investigated a) during the first five days after burn injury and b) preoperatively during the five days after surgery in three subsets of patients, including those with no preoperative and no postoperative sepsis (NN), no preoperative but postoperative sepsis (NS), and preoperative and postoperative sepsis (SS). A total of 145 patients met the selection criteria and were included in the analysis.

**Results:**

PCT levels in the first five days after burn injury were significantly higher in patients who developed at least one sepsis episode (*n* = 85) compared with patients who did not develop sepsis (*n* = 60). PCT values > 1.00 ng/mL were clearly associated with sepsis. Study participants (*n* = 145) underwent a total of 283 surgical interventions. Their distribution by preoperative/postoperative sepsis status was 142 (50.2%) in NN; 62 (21.9%) in NS; and 79 (27.9%) in SS. PCT values exhibited a parallel course in the three groups that peaked on the second postoperative day and returned to preoperative levels on the third day or later. The lowest PCT values were found in NN, and the highest values were observed in SS; the NS values were intermediate.

**Conclusions:**

PCT kinetics coupled with a clinical examination may be helpful for sepsis diagnosis during the first days after burn injury and burn surgery.

**Electronic supplementary material:**

The online version of this article (10.1186/s12871-018-0585-6) contains supplementary material, which is available to authorized users.

## Background

An early diagnosis of sepsis is of the utmost importance for the correct management of burn patients because it has a marked impact on treatment outcomes and survival [1]. Sepsis can lead to multiple organ dysfunction syndrome (MODS), which is the cause of most deaths in burn units [2]. Therefore, a prompt sepsis diagnosis and the immediate initiation of antimicrobial therapy are needed to reduce morbidity and mortality. However, the unnecessary administration of antimicrobials is often associated with adverse effects, increased costs and the emergence and spread of antimicrobial resistance.

It is clinically difficult to identify patients who are developing sepsis because the overwhelming systemic inflammatory response triggered by burn trauma mimics the signs and symptoms of sepsis [3]. A definitive diagnosis of sepsis requires microbiological cultures, but the results are not available for 24 to 48 h, and false negative results are found in 20–30% of cases. Therefore, the development of complementary tools for sepsis diagnosis, such as the use of biomarkers, is necessary [4].

Biomarkers and their kinetics may aid the clinical examination in the differentiation of infectious from non-infectious inflammatory responses [5, 6]. Numerous sepsis biomarkers are described in the literature [7], and procalcitonin (PCT) is one of the most studied biomarkers. PCT exhibits the best discriminative power of all of the biomarkers that are available at most hospital facilities [8, 9]. Thyroid C cells primarily secrete PCT in healthy subjects, and it is barely detected in blood (< 0.01 ng/mL). Many other cell types (liver, kidney, adipocytes, etc.) secrete PCT in response to direct or indirect infectious stimuli during septic episodes, and it is massively released into the bloodstream at concentrations that reach 1000 times its normal values [10]. Increased PCT is noticeable 2–4 h after sepsis onset and peaks at 24–48 h. PCT levels decrease by 50% every 1–1.5 days (half-life) when the infectious process is controlled [11]. PCT levels are highly correlated with bloodstream infections [12], and a recent meta-analysis demonstrated that elevated PCT levels and PCT non-clearance were related with an increased risk of sepsis and a higher mortality rate [13]. PCT is accurate for sepsis diagnosis, and its kinetics exhibit good correlation with sepsis severity [14]. Therefore, PCT is recommended in diverse clinical settings, including the exclusion of a bacterial cause in lower respiratory infections [15] as well as the diagnosis, stratification, prognosis [16, 17] and antimicrobial administration guidance in septic patients [18] and the diagnosis of postoperative infections [19, 20]. However, the utility of PCT in burn patients was questioned because of the high rate of false-positive results from the systemic inflammatory response induced by burn injury and subsequent surgical interventions [21, 22].

The present study evaluated PCT kinetics after a burn episode and the surgical intervention(s) needed for its treatment to assess its utility in the differential diagnosis between septic and non-septic inflammatory responses.

## Methods

### Study plan

This retrospective observational study used clinical and laboratory data collected from the health records of all burn patients admitted to Coimbra Burns Unit (CBU), a department of Coimbra Hospital and University Centre (CHUC), a tertiary referral hospital in Portugal, between January 2011 and December 2014, who presented with a 15% or more total burn surface area (TBSA) and who underwent subsequent surgery during their hospitalization. A total of 145 patients met the selection criteria, and their data were available for analysis.

Sepsis was diagnosed according to the American Burn Association (ABA) criteria [23]: presence, in at least one of the initial five days, of a clinical suspicion of infection coupled with at least three of the following findings: temperature > 39 °C or < 36.5 °C, tachycardia > 110 beats/min, tachypnea > 25 breaths/min or minute ventilation > 12 L/min, thrombocytopenia < 100,000/mL, hyperglycaemia (untreated plasma glucose > 200 mg/dL or intravenous glucose requirement > 7 U/h over 24 h), and enteral feeding intolerance (abdominal distension or gastric residuals more than two times feeding rate or diarrhoea > 2500 mL).

Serum PCT concentrations were measured using TRACE© (time-resolved amplified cryptate emission) technology (Kryptor© PCT; Brahms© AG; Hennigsdorf, Germany).

PCT kinetics were evaluated in the first five days after burn injury in the entire study population, preoperatively and during the five days after surgery in three subsets of patients: no preoperative and no postoperative sepsis (NN), no preoperative but postoperative sepsis (NS), and preoperative and postoperative sepsis (SS).

### Statistical analysis

The maximum value of PCT on each day of the study was used for statistical analyses.

Qualitative variables (e.g., gender and mortality) are described as counts, and quantitative variables (e.g., TBSA and ABSI - Abbreviated Burn Severity Index: see Additional file 1) are described as the means and corresponding standard deviations. The number of surgical interventions and PCT values by subgroup are described as medians and interquartile ranges (IQR). Comparisons between sepsis and no sepsis groups were performed using the Mann-Whitney test for quantitative variables and the Fisher’s exact test for qualitative variables. Time comparisons of PCT levels were performed using Friedman’s test.

Receiver operating characteristic (ROC) curves and comparative analysis of the area under the curve (AUC) were performed to evaluate the discriminatory power of PCT levels on consecutive days.

Statistical analysis was performed using SPSS© 23.0 IBM© for Windows©, and a *p*-value ≤0.05 was considered significant.

## Results

Table 1 presents the primary demographic and baseline characteristics of the study population, which consisted of 84 males and 61 females. The sepsis (*n* = 85) and non-sepsis (*n* = 60) groups showed no significant differences in terms of gender or age, but they were significantly different in terms of ABSI score, TBSA and mortality.

**Table 1:** Study population

Characteristics	No Sepsis	Sepsis^#^	*p*-value
Number of patients	85	60	
Gender (male/female)	45/40	39/21	0.115
Age (years)^$^	56.49 (±18.15)	58.43(±21.89)	0.517
ABSI score^a^	7.69 (±2.82)	9.17 (±2.20)	0.000*
TBSA (%)^$^	29.97 (±19.94)	34.6 (±17.26)	0.000*
Mortality (No/Yes)	84/1	24/36	0.004*

^*^p-values < 0.05

^$^Values are Median (Q1-Q3)

^#^At least one day with sepsis in the first five days after burn episode

^a^Description in Annex I

**Fig. 1:**
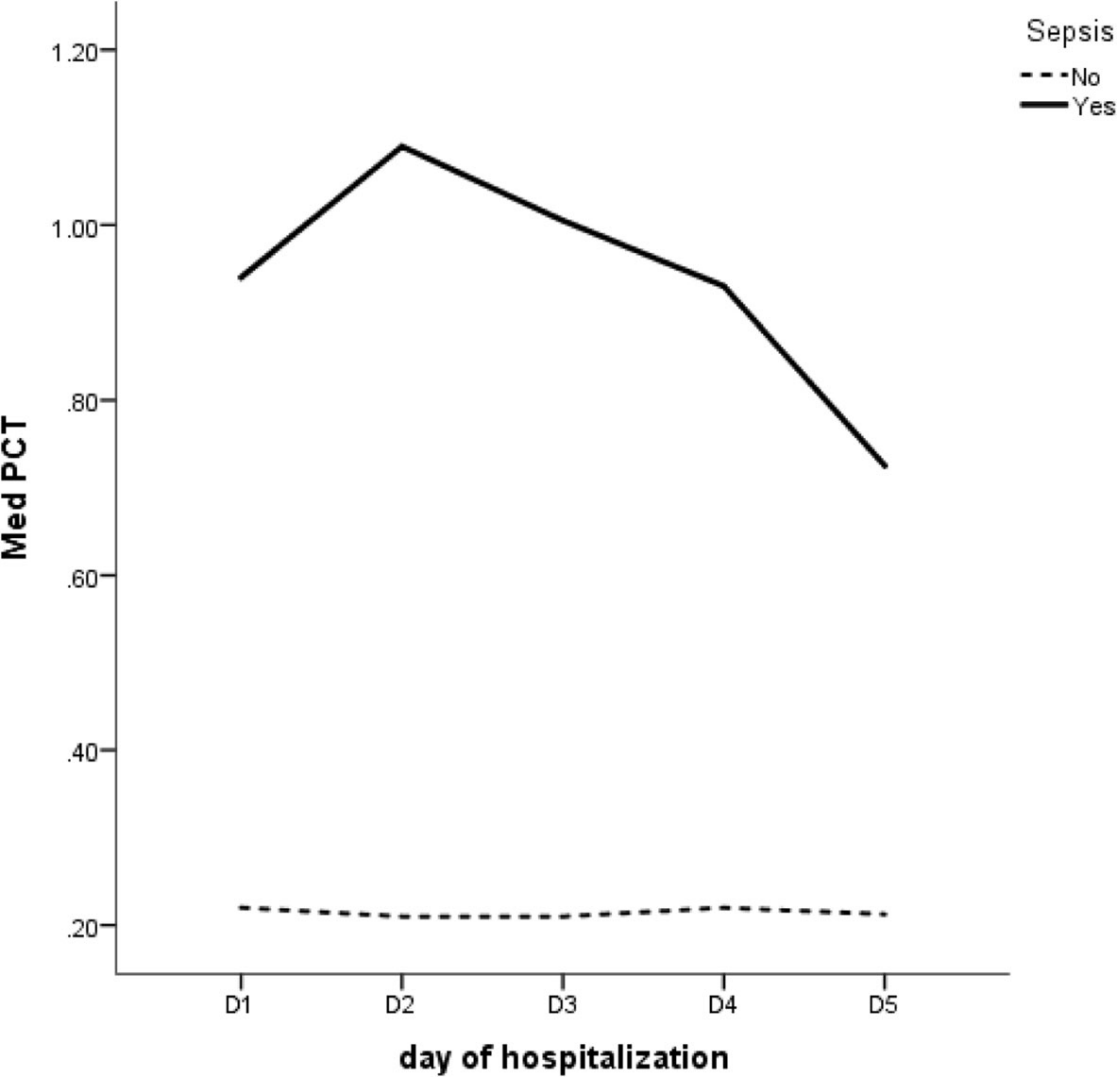
Median PCT levels observed in in the first five days after burn injury in septic (Yes) and non-septic (No) patients

**Fig. 2:**
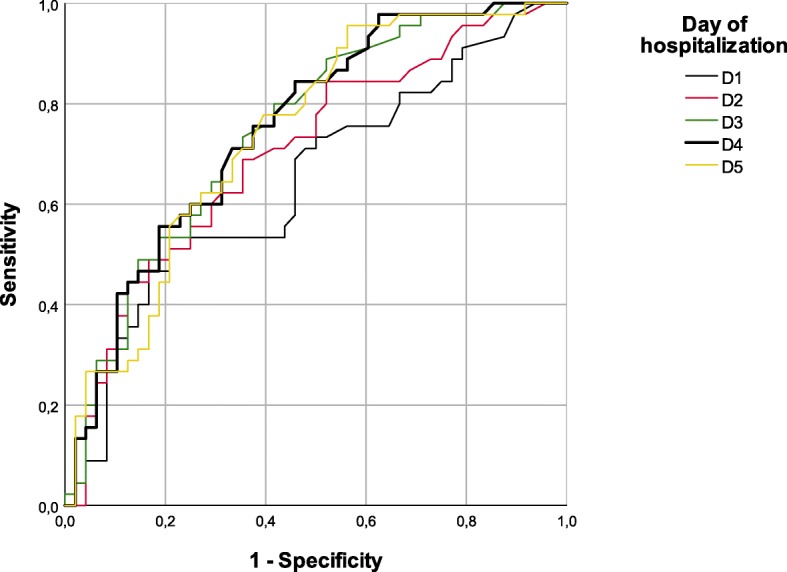
ROC Curves for the discriminatory power of PCT levels between septic and non-septic patients in the first five days after burn injury

The analysis of PCT levels during the first five days after the burn episode showed a statistically significant difference between the group of patients who developed at least one sepsis episode during that time and the group of patients who did not develop sepsis (Fig. 1). PCT values over 1.00 ng/mL were clearly associated with septic processes (*p* < 0.001, Mann-Whiney U test, Table 2). ROC curves and the AUC were performed to evaluate the discriminatory power of PCT over consecutive days. These results demonstrated that the discriminatory power of PCT levels increased over time (Table 3 and Fig. 2).

**Table 2:** Statistical analysis of PCT kinetics in the first five days after burn injury in septic and non-septic patients

Sepsis	Statistic	D1	D2	D3	D4	D5	*p*-value*
No	n	64	70	72	69	62	
	Median [IQR]	0.215 [0.090–0.578]	0.205 [0.09–0.723]	0.210 [0.08–0.668]	0.215 [0.102–0.695]	0.213 [0.118–0.618]	0.557
	n	50	53	52	53	52	
Yes	Median [IQR]	1.085 [0.188–5.440]	1.650 [0.235–4.010]	1.130 [0.335–2.920]	1.060 [0.355–2.927]	0.725 [0.340–2.105]	0.288
	*p*-value**	0.001	0.000	0.000	0.000	0.000	

*Friedman test

**Mann-Whiney U test

**Table 3:** ROC curves for the discriminatory power of PCT levels between septic and non-septic patients in the first five days after burn injury

Area Under the Curve (AUROC)			
Day	Area	Asymptotic 95% Confidence Interval	
		Lower Bound	Upper Bound
D1	0.646	0.532	0.759
D2	0.704	0.598	0.810
D3	0.746	0.647	0.845
D4	0.752	0.654	0.850
D5	0.741	0.641	0.841

All patients (*n* = 145) underwent at least one surgical intervention, with a cumulative 283 surgical interventions. Each patient was subjected to a median of three interventions, with an IQR of [2.00–5.25]. All interventions were performed under general anaesthesia and were classified as clean-contaminated. The interventions consisted primarily of escharectomies, skin autografts and flaps, and digits/limb amputations to a lesser extent.

**Fig. 3:**
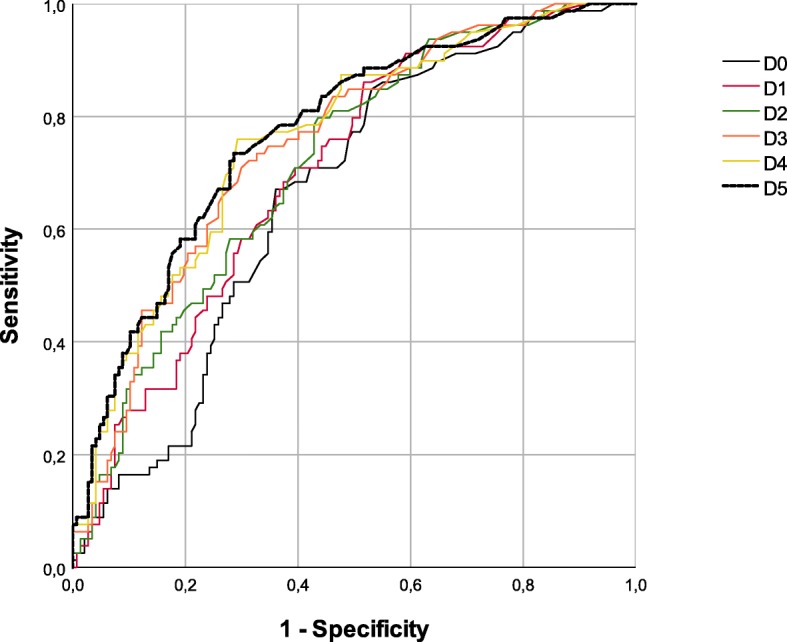
ROC Curves for the discriminatory power of PCT levels between septic and non-septic patients preoperatively and in the first five days after burn surgery

To assess the influence of surgical trauma on PCT concentrations, PCT evolution from the day before the operation (D0) until the fifth postoperative day (D5) was analysed. Differences in the time evolution of PCT between the sepsis and non-sepsis groups were statistically significant (Table 4), and the discriminatory power increased over time as shown by the ROC curve analysis (Table 5 and Fig. 3).

**Table 4:** Statistical analysis of PCT kinetics from preoperative day (D0) till the fifth postoperative day (D5) for NN, NS and SS groups

Sepsis	Statistic	D0	D1	D2	D3	D4	D5	*p*-value*
NN	n	212	208	198	186	163	149	
	Median [IQR]	0.190 [0.110–0.560]	0.200 [0.101–0.615]	0.280 [0.120–0.758]	0.223 [0.118–0.553]	0.195 [0.110–0.430]	0.180 [0.100–0.360]	0.000
NS	n	104	103	102	100	87	80	
	Median [IQR]	0.405 [0.219–0.935]	0.510 [0.240–1.360]	0.640 [0.313–1.590]	0.625 [0.283–1.438]	0.540 [0.260–1.970]	0.515 [0.273–2.045]	0.000
SS	n	74	74	74	69	65	62	
	Median [IQR]	0.653 [0.233–2.193]	0.790 [0.288–2.518]	1.115 [0.413–2.990]	0.880 [0.380–3.115]	0.710 [0.300–1.950]	0.580 [0.248–1.520]	0.000
	*p*-value**	0.000	0.000	0.000	0.000	0.000	0.000	
	Multiple comparison (*p*-value***)							
(NN,NS)		0.000	0.000	0.000	0.000	0.000	0.000	
(NN,SS)		0.000	0.000	0.000	0.000	0.000	0.000	
(NS,SS)		0.339	0.225	0.135	0.294	1	1	

*Friedman test

**Kruskal Wallis test

***Mann-Whiney U test with Bonferroni correction

**Table 5:** ROC curves for the discriminatory power of PCT levels between septic and non-septic patients preoperatively and in the first five days after burn surgery

Area Under the Curve (AUROC)			
Day	Area	Asymptotic 95% Confidence Interval	
		Lower Bound	Upper Bound
D0	0.662	0.591	0.733
D1	0.701	0.633	0.770
D2	0.717	0.649	0.784
D3	0.752	0.686	0.815
D4	0.760	0.696	0.824
D5	0.771	0.708	0.834

**Fig. 4:**
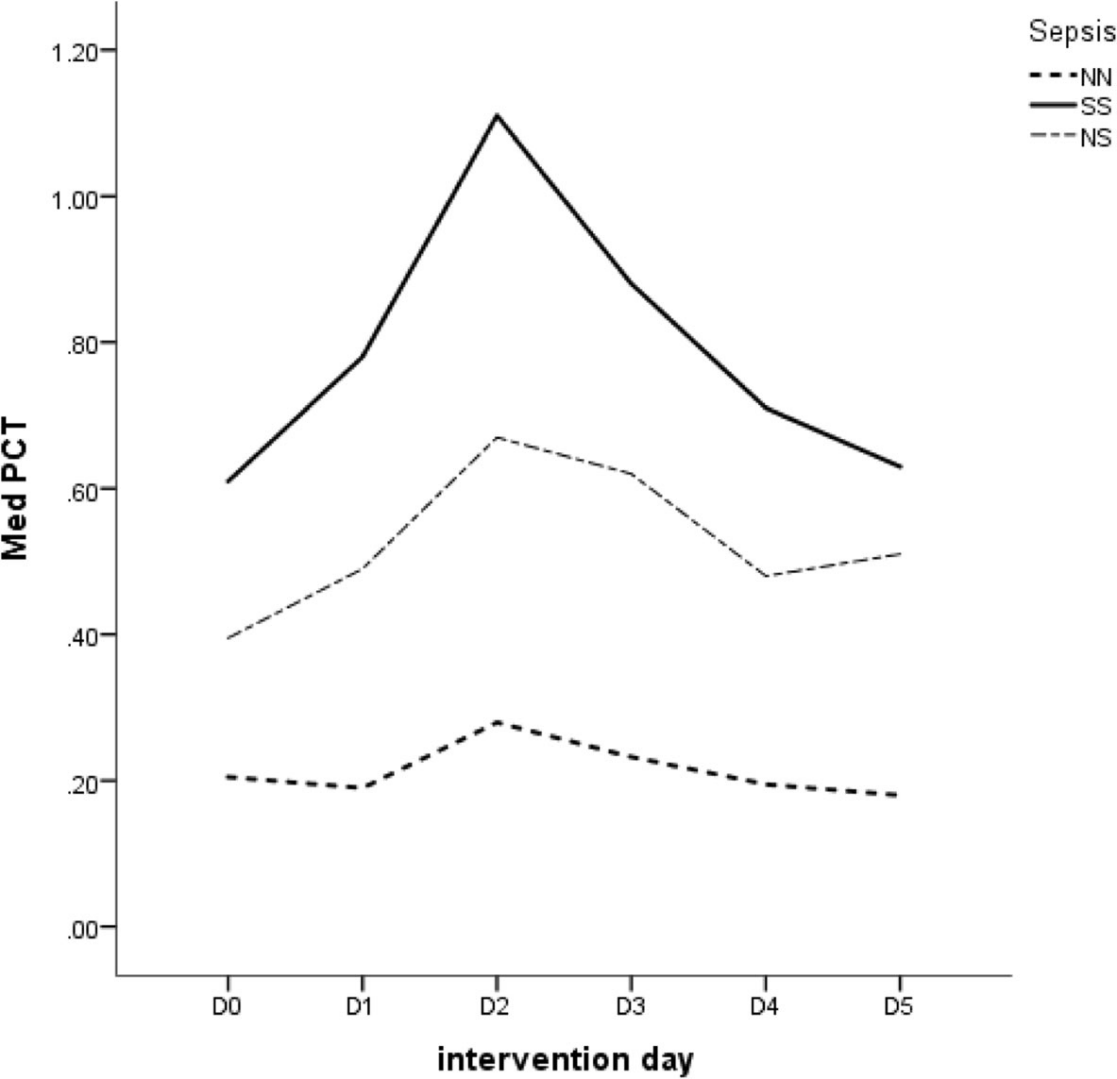
Median PCT levels observed from preoperative day (D0) till the fifth postoperative day (D5) for NN, NS and SS groups

Regarding the preoperative/postoperative sepsis status, median values had a parallel course in the three groups (Fig. 4). Values peaked in the second postoperative day and returned to preoperative levels on the third day or later. The lowest values were found in the NN group, which included 142 surgical interventions in patients without preoperative sepsis and who did not develop postoperative sepsis through D5 (50.2%). The highest values were observed in the SS group, which included 62 surgical interventions in patients with pre- and postoperative sepsis (21.9%). Group NS exhibited PCT values roughly in the middle range between the other two groups and included 79 surgical interventions in patients who did not exhibit septic processes preoperatively but developed sepsis on at least one of the five days after surgery (27.9%). The kinetics of the PCT levels within each group (Table 3) were significantly different between days after surgery in the absence (NN) or presence of sepsis (NS and SS).

## Discussion

The present study included a sample of 145 burn patients from the CBU, and PCT levels were significantly different between septic and non-septic patients during the first five days after burn injury. The results indicate that PCT values evolved in parallel with sepsis development and the antimicrobial therapy effect. In this important population, PCT consistently showed good potential to discriminate between septic and non-septic patients, particularly when frequent PCT assays were performed and when its kinetics were dynamically assessed.

To evaluate PCT performance after surgical interventions and to investigate whether surgical trauma alone could reduce the accuracy of the diagnosis of postoperative sepsis, this study included a substantial and diversified number of interventions performed in the three subsets of patients who were organized according to the existence or absence of preoperative sepsis and the development or worsening of sepsis after surgery. PCT levels increased modestly and rapidly returned to basal levels after the second postoperative day in patients with no preoperative or postoperative sepsis episodes. Patients with increased preoperative PCT values that corresponded to preoperative sepsis exhibited PCT kinetics with a higher peak on the second postoperative day, which was presumably related to the additive increment of PCT of surgical trauma. PCT values returned to the initial values when antimicrobial therapy was administered. PCT levels in patients who only developed sepsis after surgery exhibited a parallel evolution to the already septic patients but generally with lower absolute values. Therefore, PCT is useful for sepsis diagnosis in cases of surgical intervention when preoperative PCT values are known because PCT kinetics follow the same pattern of evolution in cases of sepsis as in other critical patients.

The search for sepsis biomarkers is an exciting and never-ending story [24, 25]. Diverse approaches were used to identify more precise, practical, quicker, safer and cheaper chemicals or physical changes that may indicate the urgent need and adequacy of antimicrobial therapy or its redundancy to reduce adverse events, microbial resistance and financial costs. Current research is more focused on molecular (PCR, MALDI-TOF) and/or system-based (genomics, transcriptomics, proteonomics, metabolomics) methods for sepsis diagnosis [26–29], but these techniques are not fully developed, practical or widely available.

An ideal biomarker is not developed, and the use of PCT as an early distinction between actual septic patients and patients with merely systemic inflammatory signals and symptoms during the first days after hospital admission has been largely discussed in the medical literature in the last two decades [30–35]. PCT is a useful but not ideal biomarker, particularly due to its negative predictive power [36], which led to its inclusion in algorithms for sepsis management [37, 38]. The use of serial measurements instead of a single observation reinforces the predictive power of PCT and reduces the risks of false negatives and false positives [39–43]. The same considerations are valid for the use of PCT in the investigation of suspected postoperative sepsis [44], which is currently performed after many types of surgical procedures [45–49].

PCT accuracy in burn patients is controversial [21, 22, 50, 51]. Burn patients are generally excluded from sepsis studies and clinical trials based on the simplistic assumption that PCT levels are always elevated in burn patients as a result of the non-septic inflammatory systemic response related to burn trauma. However, several studies consistently demonstrated different PCT kinetics in burn patients based on the presence or absence of systemic infection [52–55]. Three recent meta-analysis also validated the use of PCT for sepsis diagnosis in these patients [56–58]. PCT evolution is predictable in both cases, and it provides a reliable means to identify septic processes, which was first referred to by von Heimburg et al. in 1998 [59]. The immediate inflammatory burst elevates PCT levels after burn injury, independent of infection, and grossly correlates with TBSA, but it rarely surpasses 2.0 ng/mL [60, 61]. The maximum PCT value is reached within 24–48 h in the absence of sepsis and returns to normal values (1.0–1.5 ng/mL or less) by the end of the third day. PCT levels continue increasing in the presence of sepsis and rapidly reach values greater than 5–100 ng/mL. PCT levels only diminish with antimicrobial therapy or terminal immunosuppression, as observed in other forms of severe trauma [62]. Lavrentieva et al. analysed a sample of 145 burn patients and found increased PCT levels during the first 24 h after a burn episode, which subsided in non-septic patients and continued increasing in septic patients. These authors demonstrated an inverse relationship of PCT level tendency with antimicrobial therapy efficacy. They proposed a cut-off of 1.5 ng/mL to distinguish between septic and non-septic patients [63]. Egea-Guerrero et al. [64] and Kim et al. [65] found the same PCT kinetics and approximate cutoffs.

PCT exhibited a similar kinetics pattern after surgical intervention [66], but preoperative PCT levels must be known to use these levels to discriminate between the postoperative physiological inflammatory response and postoperative sepsis. Preoperative PCT levels are related to the presence or absence of an ongoing sepsis process and possible ongoing antimicrobial therapy, which naturally influence baseline values [67]. To the best of our knowledge, the present study is the first study to specifically address PCT kinetics after surgical procedures in burn patients and demonstrate that this biomarker maintains its performance in this particular set of patients, even in the presence of preoperative sepsis.

PCT levels coupled with rigorous clinical monitoring and blood cultures as the diagnostic cornerstone [68] may help confirm or exclude sepsis in patients during the acute phase after burn trauma and ascertain the presence of postoperative sepsis in burn patients. Neither immunodepression [69] nor corticotherapy [70] affected the diagnostic performance of PCT, as opposed to other biomarkers, and PCT also distinguishes contamination from actual bloodstream infection [71]. The use of PCT dosing may inclusively reduce healthcare costs and avoid the superfluous use of antimicrobials and consequent increments on microbial resistance [72, 73].

The present study also presents some limitations. First, it was a single-centre, retrospective observational study, and the results require confirmation using prospective multicentre trials. Second, the precise influence of antimicrobial therapy in septic patients could not be evaluated because of ethical considerations that naturally prevent antimicrobial denial in face of septic episodes. Third, subgroup analyses according to the total burned surface area (TBSA) and the severity of patients’ attainment, for instance, using the Abbreviated Burn Severity Index (ABSI), was not performed. However, the use of defined and internationally accepted criteria for the clinical suspicion of burn sepsis, the homogeneity of therapeutic procedures, and the use of a standard methodology for the collection, recording and statistical analysis of the data are clearly strengths of the present study.

## Conclusion

The present study was performed in 145 burn patients who underwent a high and diversified number of surgical interventions. The results allow us to conclude that 1) PCT kinetics may aid in the differential diagnosis between true sepsis and the normal inflammatory response to burn trauma in the first days after burn injury; and 2) PCT kinetics may be used to identify postoperative sepsis in burn patients who undergo surgical interventions during their stay in burn units.

Prospective multicentre studies in adult and paediatric burn patients are needed to confirm these findings and compare PCT and other biomarkers in these contexts.

## Additional file

Additional file 1: Abbreviated Burn Severity Index. (DOCX 17 kb)

## Abbreviations

ABA: American Burn Association
ABSI: Abbreviated Burn Severity Index
AUC: Area Under the Curve
CBU: Coimbra Burn Unit
CHUC: Coimbra University Hospital Centre (Centro Hospitalar e Universitário de Coimbra)
CIDMA: Center for Research and Development in Mathematics and Applications, Department of Mathematics, University of Aveiro
CIOMS: Council for International Organizations of Medical Sciences
iBiMED: Institute for Biomedicine, Department of Mathematics, University of Aveiro
IBM: International Business Machines
IQR: Interquartile Range
MALDI-TOF: Matrix-Assisted Laser Desorption Ionization – Time of Flight
MedinUP: Department of Pharmacology and Therapeutics, Faculty of Medicine, University of Porto
MODS: Multiple Organ Dysfunction Syndrome
PCR: Polymerase Chain Reaction
PCT: Procalcitonin
ROC: Receiver Operating Characteristic
SACS: Autonomous Section of Health Sciences (Secção Autónoma de Ciências da Saúde), University of Aveiro
SPSS: Statistical Package for the Social Sciences
TBSA: Total Burn Surface Area
TRACE: Time-Resolved Amplified Cryptate Emission

## References

[1] Nunes Lopez O, Cambiaso-Daniel J, Branski LK, Norbury WB, Herndon DH (2017). Predicting and managing sepsis in burn patients: current perspectives. Ther Clin Risk Manag.

[2] Greenhalgh DG (2017). Sepsis in the burn patient: a different problem than sepsis in the general population. Burns & Trauma.

[3] Norbury W, Herndon DH, Tanksley J, Jeschke M (2016). Infections in burns. Surg Infect.

[4] Jin H (2014). Liu z, Xiao Y, fan X et al. prediction of sepsis in trauma patients. Burns & Trauma.

[5] Ruíz-Caestilla M, Roca O, Masclans JR, Barret JP (2016). Recent advances in biomarkers in severe burns. Shock.

[6] Arora R, Campbell JP, Simon G, Sahni N (2017). Does serum procalcitonin aid in the diagnosis of bloodstream infection regardless of whether patients exhibit the systemic inflammatory response syndrome?. Infection.

[7] Pierrakos C, Vincent JL (2010). Sepsis biomarkers: a review. Crit Care.

[8] Charles PE, Kus E, Aho S, et al. Serum procalcitonin for the early recognition of nosocomial infection in the critically ill patients: a preliminary report. BMC Infect Dis. 2009;9(49) 10.1186/1471-2334-9-4. 10.1186/1471-2334-9-49 PMC2679028 19386110

[9] Marik PE (2014). Don’t miss the diagnosis of sepsis!. Crit Care.

[10] Vincent JL, van Nuffelen M, Lelubre C, Mancini N (2015). Host response biomarkers in sepsis: the role of procalcitonin. Sepsis: Diagnostic Methods and Protocols, Methods Mol Biol.

[11] Meisner M (2014). Update on procalcitonin measurements. Annals of Laboratory Medicine.

[12] Hoeboer SH, van der Geest PJ, Nieboer D, Groeneveld AB (2015). The diagnostic accuracy of procalcitonin for bacteraemia: a systematic review and meta-analysis. Clin Microbiol Infect.

[13] Liu D, Su L, Han G (2015). Prognostic value of procalcitonin in adult patients with sepsis: a systematic review and meta-analysis. PLoS One.

[14] Vincent JL, Teixeira L (2014). Sepsis biomarkers. Value and limitations. Am J Respir Crit Care Med.

[15] Schuetz P, Müller B, Christ-Crain M, Stolz D (2013). Procalcitonin to initiate or discontinue antibiotics in acute care respiratory tract infections. Evid-Based Child Health.

[16] Schuetz P, Maurer P, Punjabi V, Desai A (2013). Procalcitonin decrease over 72 hours in US critical care units predicts fatal outcome in sepsis patients. Crit Care.

[17] Ryu JA, Yang JH, Lee D, Suh GY (2015). Clinical uselfulness of procalcitonin and C-reactive protein as outcome predictors in critically patients with severe sepsis and septic schock. PLoS One.

[18] Bouadma L, Luyt CE, Tubach F, Cracco C (2010). Use of procalcitonin to reduce patients’ exposure to antibiotics in intensive care units (PRORATA trial): a multicentre randomised controlled trial. Lancet.

[19] Mokart D, Merlin M, Sannini A, Brun JP (2005). Procalcitonin, Interleucin 6 and systemic inflammatory response syndrome (SIRS): early markers of sepsis after major surgery. Br J Anaesth.

[20] Meyer ZC, Schreinemakers JM, de Waal RA, van der Laan L (2015). Searching for predictors of surgical complications in critically ill surgery patients in the intensive care unit: a review. Surg Today.

[21] Seoane L, Pertega S, Galeiras R, Astola I, Bouza T (2014). Procalcitonin in the burn unit and the diagnosis of infection. Burns.

[22] Honore PM, Spapen HD (2016). The struggle to differentiate inflammation from infection in severely burned patients: time to send better biomarkers into the arena?. Crit Care.

[23] Greenhalgh DG, Saffle JR, Holmes JH (2007). American burn association consensus conference to define sepsis and infection in burns. J Burn Care Res.

[24] Kibe S (2011). Diagnostic and prognostic biomarkers of sepsis in critical care. J Antimicrob Chemother.

[25] Long B, Koyfman A (2017). Ready for prime time? Biomarkers in Sepsis. Emerg Med Clin North Am.

[26] Sandquist M, Wong HR (2014). Biomarkers of sepsis and their potential value in diagnosis, prognosis and treatment. Expert Rev Clin Immunol.

[27] Yan S, Tsurumi A, Que YA, Ryan CM (2015). Prediction of multiple infections after severe burn trauma: a prospective cohort control. Ann Surg.

[28] Hazeldine J, Hampson P, Lord JM (2016). The diagnostic and prognostic value of systems biology research in major traumatic and thermal injury: a review. Burns & Trauma.

[29] David VL, Ercisli MF, Rogobete AF, Boia ES (2017). Early prediction of sepsis incidence in critically ill patients using specific genetic polymorphisms. Biochem Genet.

[30] Harbarth S, Holeckova K, Froidevaux C, Pittet D (2001). Diagnostic value of procalcitonin, interleukin-6 and interleukin-8 in critically ill patients admitted with suspected sepsis. Am J Respir Crit Care Med.

[31] Tang BM, Eslick GD, Craig JC, McLean AS (2007). Accuracy of procalcitonin for sepsis diagnosis in critically ill patients: systemic review and meta-analysis. Lancet Infect Dis.

[32] Wacker C, Prkno A, Brunkhorst FM, Schlattmann P (2013). Procalcitonin as a diagnostic marker of sepsis: a systematic review and meta-analysis. Lancet Infect Dis.

[33] Garnacho-Montero J, Huici-Moreno MJ, Gutierrez-Pizarraya A, López I (2014). Prognostic and diagnostic value of eosinopenia, C-reactive protein, procalcitonin and circulating cell-free DNA in critically ill patients admitted with suspicion of sepsis. Crit Care.

[34] Lippi G, Montagnana M, Balboni F, Bellone A (2017). Academy of emergency medicine and Care-Society of Clinical Biochemestry and Clinical Molecular Biology consensus recommendations for clinical use of sepsis biomarkers in the emergency department. Emergency Care Journal.

[35] Sager R, Kutz A, Mueller B, Schuetz P. Procalcitonin-guided diagnosis and antibiotic stewardship revisited. BMC Med. 2017;15(15) 10.10186/s12916-017-0795-7. 10.1186/s12916-017-0795-7 PMC5259962 28114931

[36] Vincent JL (2016). The clinical challenge of sepsis identification and monitoring. PLoS Med.

[37] de Jong E, van Oers JA, Beishuizen A (2016). Efficacy and safety of procalcitonin guidance in reducing the duration of antibiotic treatment in critically ill patients: a randomised, controlled, open-label trial. Lancet Infect Dis.

[38] Schuetz P, Chiappa V, Briel M, Greenwald JL (2011). Procalcitonin algorithms for antibiotic therapy decisions: a systematic review of randomized controlled trials and recommendations for clinical algorithms. Arch Intern Med.

[39] Molnár Z, Bogár L (2006). Let’s go dynamic with procalcitonin. Crit Care Med.

[40] Vincent JL (2014). Sepsis biomarkers – values and limitations. Am J Respir Crit Care Med.

[41] Lipinska-Gediga M, Mierzchata-Pasierb M, Durek G (2016). Procalcitonin kinetics – prognostic and diagnostic significance in septic patients. Arch Med Sci.

[42] Trasy D, Molnar Z (2017). Procalcitonin – assisted antibiotic strategy in sepsis. J Int Fed Clin Chem.

[43] Schuetz P, Robert Birkhahn R, Robert Sherwin R, Jones AE (2017). Serial procalcitonin predicts mortality in severe sepsis patients: results from the multicenter procalcitonin monitoring sepsis (MOSES) study. Crit Care Med.

[44] Meisner M, Tschaikowsky K, Hutzler A, Schick C, Schüttler J (1998). Postoperative plasma concentrations of procalcitonin after different types of surgery. Intensive Care Med.

[45] Sponholz C, Sakr Y, Reinhart K, Brunkhorst F (2006). Diagnostic value and prognostic implications of serum procalcitonin after cardiac surgery: a systematic review of the literature. Crit Care.

[46] Friederichs J, Hutter M, Hierholzer C, Novotny A (2013). Procalcitonin as a predictor of successful surgical treatment of severe necrotizing soft tissue infections. Am J Surg.

[47] Muñoz JL, Ruiz-Tovar J, Miranda E, Berrio DL (2016). C-reactive protein and procalcitonin as early markers of septic complications after laparoscopic sleeve gastrectomy in morbidly obese patients with an enhanced recovery after surgery program. J Am Coll Surg.

[48] Varetto G, Castagno C, Trucco A, Frola E (2016). Serum procalcitonin as a valuable diagnostic tool in the early detection of infectious complications after abdominal aortic repair. Ann Vasc Surg.

[49] Spoto S, Valeriani E, Caputo D, Cella E (2018). The role of procalcitonin in the diagnosis of bacterial infection after major abdominal surgery - advantage from daily measurement. Medicine.

[50] Bargues L, Chancerelle Y, Catineau J, Jault P, Carsin H (2007). Evaluation of serum procalcitonin concentration in the ICU following severe burn. Burns.

[51] Schultz L, Walker SA, Elligsen M, Walker SE (2013). Identification of predictors of early infection in acute burn patients. Burns.

[52] Lavrentieva A, Kontakiotis T, Lazaridis L (2007). Inflammatory markers in patients with severe burn injury: What is the best indicator of sepsis. Burns.

[53] Barati M, Alinejad F, Bahar MA (2008). Comparison of WBC, ESR, CRP and PCT serum levels in septic and non-septic burn cases. Burns.

[54] Mokline A, Garsallah L, Rahmani I, Jerbi K (2015). Procalcitonin: a diagnostic and prognostic biomarker of sepsis in burned patients. Ann Burns Fire Disasters.

[55] Cabral L, Afreixo V, Santos S, Almeida L, Paiva JA (2017). Procalcitonin for the early diagnosis of sepsis in burn patients; a retrospective study. Burns.

[56] Mann EA, Wood GL, Wade CE (2011). Use of procalcitonin for the detection of sepsis in the critically ill burn patient: a systematic review of the literature. Burns.

[57] Ren H, Li Y, Han C, Hu H (2015). Serum procalcitonin as a diagnostic biomarker for sepsis in burn patients: a meta-analysis. Burns.

[58] Cabral, V Afreixo, L Almeida, Paiva JA. The use of procalcitonin (PCT) for diagnosis of sepsis in burn patients: a meta-analysis. PLoS One 2016;11: e0168475. DOI:10.1371/journal.pone.0168475. 10.1371/journal.pone.0168475 PMC5179235 28005932

[59] von Heimburg D, Stieghorst W, Khorram-Sefat R, Pallua N. Procalcitonin – a sepsis parameter in severe burn injuries. Burns 1998;24:745–750. PMID:9915676. 10.1016/s0305-4179(98)00109-0 9915676

[60] Sachse C, Machens HG, Felmerer G, Berger A, Henkel E (1999). Procalcitonin as a marker for the early diagnosis of severe infection after thermal injury. J Burn Care Rehabil.

[61] Ciriello V, Gudipati S, Stavrou PZ, Kanakaris NK (2013). Biomarkers predicting sepsis in polytrauma patients: current evidence. Injury.

[62] Billeter A, Turina M, Seifert B, Mica L (2009). Early serum procalcitonin, interleukin-6 and 24-hour lactate clearance: useful indicators of septic infections in severely traumatized patients. World J Surg.

[63] Lavrentieva A, Papadopoulou S, Kioumis J, Kaimakamis E, Bitzani M (2012). PCT as a diagnostic and prognostic tool in burn patients - whether time course has a role in monitoring sepsis treatment. Burns.

[64] Egea Guerrero JJ, Martínez-Fernandez C, Rodríguez-Rodríguez A, Bohorquez-López A (2015). The utility of C-reactive protein and procalcitonin for sepsis diagnosis in critically burned patients: a preliminary study. Plastic Surgery.

[65] Kim HS, Yang HT, Hur J, Chun W (2012). Procalcitonin levels within 48 hours after burn injury as a prognostic factor. Ann Clin Lab Sci.

[66] Dahaba AA, Hagara B, Fall A, Rehak PH (2006). Procalcitonin for early prediction of survival outcome in postoperative critically ill patients with severe sepsis. Br J Anaesth.

[67] Ljungström L, Pernestig A-K, Jacobsson G, Andersson R, Usener B, Tilevik D (2017). Diagnostic accuracy of procalcitonin, neutrophil-lymphocyte count ratio, C-reactive protein, and lactate in patients with suspected bacterial sepsis. PLoS One.

[68] Riedel S (2012). Procalcitonin and the role of biomarkers in the diagnosis and management of sepsis. Diagn Microbiol Infect Dis.

[69] Bele N, Darmon M, Coquet I, Feugeas JP (2011). Diagnostic accuracy of procalcitonin in critically ill immunocompromised patients. BMC Infect Dis.

[70] Schuetz P, Albrich W, Christ-Crain M, Chastre J, Mueller B (2010). Procalcitonin for guidance of antibiotic therapy. Expert Rev Anti-Infect Ther.

[71] Hattori T, Nishiyama H, Kato H, Ikegami S (2014). Clinical value of procalcitonin for patients with suspected bloodstream infections. Am J Clin Pathol.

[72] Steuten L, Mantjes G (2016). Economic value of procalcitonin guidance. Lancet Infect Dis.

[73] Balk RA, Kadri SS, Cao Z (2017). Effect of procalcitonin testing on health-care utilization costs in critically ill patients in the United States. Chest.

