# Supplementary material for: Procalcitonin kinetics after burn injury and burn surgery in septic and non-septic patients – a retrospective observational study
Source: BMC Anesthesiol. 2018 Sep 5;18:122. doi: 10.1186/s12871-018-0585-6 (PMC6123981; doi:10.1186/s12871-018-0585-6)
Supplement: Supplementary file 1 — Abbreviated Burn Severity Index. (DOCX 17 kb) [file 12871_2018_585_MOESM1_ESM.docx]

**Additional File 1 - Abbreviated Burn Severity Index**

| Abbreviated Burn Severity Index | | |
| --- | --- | --- |
| Parameter | Finding | Points |
| Gender | Female | 1 |
|  | Male | 0 |
| Age (years) | 0-20 | 1 |
|  | 21-40 | 2 |
|  | 41-60 | 3 |
|  | 61-80 | 4 |
|  | 81-100 | 5 |
| Inhalation injury | Present | 1 |
|  | Absent | 0 |
| Full-thickness burn | Present | 1 |
|  | Absent | 0 |
| Total burned surface area | 1-10 | 1 |
|  | 11-20 | 2 |
|  | 21-30 | 3 |
|  | 31-40 | 4 |
|  | 41-50 | 5 |
|  | 51-60 | 6 |
|  | 61-70 | 7 |
|  | 71-89 | 8 |
|  | 81-90 | 9 |
|  | 91-100 | 10 |
| Prediction of outcome according to ABSI score | | |
| ABSI | Mortality risk | Probability of survival (%) |
| 2-3 | Very low | ≥ 99 |
| 4-5 | Moderate | 98 |
| 6-7 | Moderately severe | 80-90 |
| 8-9 | Serious | 50-70 |
| 10-11 | Severe | 20-40 |
| ≥ 12 | Maximum | ≤ 10 |

**References**

1. Tobiasen J, Hiebert JM, Edlich RF. The abbreviated burn severity in[dex.](https://www.ncbi.nlm.nih.gov/pubmed/7073049)

Ann Emerg Med 1982;11:260-2.

PMID:7073049

1. Tahir SM, Memom AR, Kumar M, Ali SA. Prediction of mortality after major burn: physiological versus biochemical measures.

Wounds 2009;21:177–182

PMID:25903515
